# Bridging the gaps in interventional cardiology disparities—socioeconomic, geographic, and political inequalities

**DOI:** 10.1093/ehjopen/oeaf151

**Published:** 2025-11-18

**Authors:** Marta Kaluzna-Oleksy, Giulia Massiero, Salvatore De Rosa, Marta Bujak, Rania Hammami, Omeir Kadri, Mila Kovacevic, Rita Caldeira de Rocha, José Luis Leiva Pons, Marie-Claude Morice, Alaide Chieffo

**Affiliations:** 1st Department of Cardiology, University of Medical Sciences in Poznan, Długa ½ Street, Poznan 61-848, Poland; Department of Cardiac, Thoracic, Vascular Sciences and Public Health, University of Padua Medical School, 35128 Padua, Italy; Department of Medical and Surgical Sciences, Magna Graecia University of Catanzaro, 88100 Catanzaro, Italy; Division of Cardiology and Structural Heart Diseases, Medical University of Silesia in Katowice, 40-635 Katowice, Poland; Faculty of Medicine of Sfax, Military University Hospital of Sfax, University of Sfax, 3029 Sfax, Tunisia; Department of Cardiology, Heart and Vascular Center, Rheinlandklinikum Neuss GmbH, 41540 Dormagen, Germany; Faculty of Medicine, University of Novi Sad, 21000 Novi Sad, Serbia; Institute for Cardiovascular Diseases of Vojvodina, 21204 Sremska Kamenica, Serbia; Cardiologist Serviço de Cardiologia, Hospital do Espírito Santo, 7000-811 Évora, Portugal; Autonomous University of San Luis Potosi, 78290 San Luis Potosi, Mexico; Centre Europeen de Recherche Cardiovasculaire, 7 rue du Théâtre, 91300 Massy, France; Interventional Cardiology Unit, IRCCS San Raffaele Scientific Institute, 20132 Milan, Italy

**Keywords:** Interventional cardiology, Healthcare disparities, Health equity, Socioeconomic and geographic barriers, Telemedicine

## Abstract

Despite significant advancements in interventional cardiology, including PCI, TAVI, and other structural heart interventions, access to these life-saving procedures remains uneven across the globe. This viewpoint highlights how socioeconomic, geographic, and political disparities impact clinical decision-making, outcomes, and professional well-being. Drawing from real-world experience and health systems analysis, the article explores the multifaceted barriers that hinder equitable care—ranging from health literacy and rural access to workforce shortages and regulatory or reimbursement challenges. It further discusses the psychological burden on clinicians caused by moral distress and limited resources. Potential solutions, including telemedicine, decentralized training, public awareness campaigns, and policy advocacy, are proposed to bridge the gap and promote a more just and inclusive landscape in interventional cardiology.

## Graphical Abstract

**Graphical Abstract:**
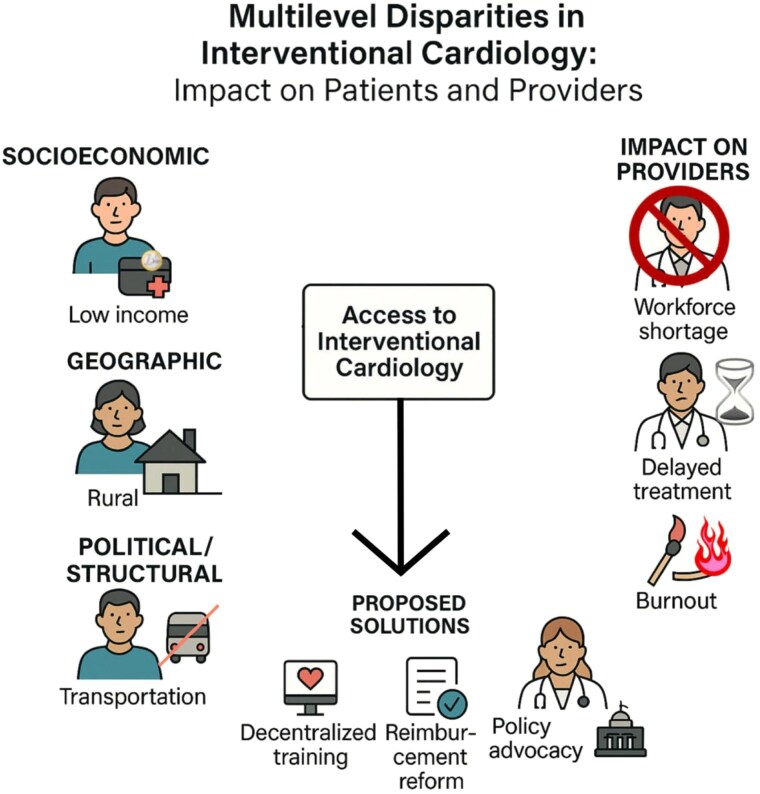

## Introduction

Over the past decades, interventional cardiology has witnessed remarkable advancements that have transformed the management of cardiovascular disease. Procedures such as percutaneous coronary intervention (PCI), transcatheter aortic valve implantation (TAVI), and other structural interventions have significantly improved survival, quality of life, and long-term outcomes for millions of patients worldwide. These innovations, supported by advances in imaging, pharmacotherapy, and device engineering, have redefined standards of care and made previously inoperable conditions treatable.

Despite these achievements, equal access to such life-saving procedures remains far from universal. Socioeconomic constraints, geographic limitations, and political or systemic healthcare barriers continue to influence who receives timely and appropriate interventional care.^1,2^ In many regions—particularly low-resource, rural, or underserved communities—patients are still unable to benefit fully from these medical breakthroughs.^3^ Unfortunately, disparities have become more prominent very recently for multiple reasons, including post-pandemic healthcare systems strain, increased awareness of equity in global healthcare and wide spreading military conflicts.^4^

The purpose of this viewpoint article is to explore how these disparities manifest in clinical practice and to examine their real-world consequences on decision-making and patient outcomes. Drawing on the perspective of practicing physicians, this paper highlights the complex interplay between systemic inequities and frontline cardiovascular care—while identifying potential strategies to bridge these gaps and promote more equitable interventional cardiology worldwide.

## Socioeconomic barriers

### Income and financial Status

Socioeconomic disparities remain a critical determinant in the accessibility and outcomes of interventional procedures. Individuals from low-income backgrounds are significantly more likely to experience delays or even denial of life-saving interventions such as PCI, and while disparities have diminished for established ACS procedures, they persist for drug-eluting stent (DES) implantation, with mortality differences strongly linked to delays in primary PCI, highlighting a critical target for intervention.^5^ Economic disparities also exist in the utilization of structural heart diseases therapies even within the United States, despite comparable adjusted in-hospital outcomes across income groups, underscoring the need for multifaceted strategies to close these gaps.^6^ Limited financial resources contribute to lower utilization rates due to unaffordable out-of-pocket expenses for diagnostics, co-pays for hospital procedures, and essential medications. In addition, indirect costs—such as transportation to tertiary care centers—further compound the burden, especially for rural or marginalized populations, often resulting in deferred care until symptom escalation or emergent presentation.^7^

### Insurance coverage disparities

The presence and quality of health insurance further stratify access to interventional cardiology. Uninsured or underinsured patients are statistically less likely to undergo timely diagnostic catheterization or advanced therapies like drug-eluting stents or TAVI.^8^ Even for patients with coverage, administrative obstacles such as prior authorization requirements or the obligation to go to specific centers for the costs to be covered can delay elective procedures, leading to disease progression or suboptimal outcomes. The variability in policy coverage between private and public insurers often dictates the type and timing of care, disproportionately disadvantaging economically vulnerable groups.

### Health literacy and patient engagement

Health literacy—a factor often intertwined with socioeconomic status—profoundly influences patient engagement in cardiovascular care.^9^ Individuals with lower education levels may struggle to recognize early symptoms of ischemia or heart failure, leading to delayed presentation and reduced eligibility for interventional procedures. This difficulty is particularly evident among women and individuals from minority ethnic backgrounds, in whom symptom recognition is especially challenging.^10^ Furthermore, socioeconomic barriers frequently hinder participation in post-procedural cardiac rehabilitation or long-term follow-up. Financial limitations, inflexible work schedules, and transportation deficits contribute to poor adherence, which can ultimately compromise long-term outcomes and widen health inequities.^11,12^ Briefly, lower education levels affect patients’ understanding of symptoms and urgency (*Table 1*).

**Table 1:** Summary of socioeconomic barriers in interventional cardiology

Barrier Category	Specific Issues	Impact on Care
Income and Financial Status	High out-of-pocket costs, transportation challenges	Delayed or denied access to PCI/TAVI
Insurance Disparities	Lack of coverage, prior authorization delays	Fewer advanced procedures, increased wait times
Health Literacy and Engagement	Low awareness of symptoms, limited understanding of follow-up and rehab importance	Late presentation, underutilization of cardiac rehab

## Geographic disparities

### Urban-rural divide

The unequal distribution of catheterization labs and high-volume cardiac centers accentuates geographic disparities, heavily favoring urban centers.^13^ Rural populations have notably reduced access to PCI, leading to worse outcomes in acute myocardial infarction (MI). For example, rural MI patients undergo catheterization, PCI or coronary artery bypass graft (CABG) surgery significantly less often (49.7% vs. 63.6% for catheterization; *P* < 0.001, 42.1% vs. 45.7% for PCI; *P* < 0.001, 9.0% vs. 10.2% for CABG; *P* < 0.001 respectively), within 30-days with 30-day mortality hazard ratio of 1.10 in rural settings.^13^ Another registry showed rural in-hospital ST-segment myocardial infarction (STEMI) inpatients had PCI rates of just 3.8% vs. urban 13.8%; *P* < 0.01, with a one-year mortality of 68.6% vs. 58.9%; *P* < 0.01.^3^

### Workforce maldistribution

The shortage of interventional cardiologists in underserved areas makes it hard to sustain on-call services and maintain procedural competence. Operators in rural settings often fall below the recommended 50 PCIs/year threshold, which is linked to increase in-hospital mortality—low-volume operators have a 1.16-fold higher adjusted mortality than high-volume peers.^14^ This compounds burnout and diminishes clinical quality in rural hospitals.^15^

### Emergency network gaps

STEMI care in rural or low-resource regions lacks the structured coordination seen in urban networks. Many regions lack 24/7 PCI-capable facilities and formalized EMS protocols, contributing to delayed reperfusion and poor outcomes.^16,17^ Moreover, in some regions the density of catheterization laboratories falls far below European recommendations of 1.67–2 per million population—for instance, Indonesia averages only about 1.1–1.2 cath labs per million, while the Philippines similarly reports a critically low and uneven distribution.^18^ Reports from quality improvement initiatives in rural hospitals confirm persistent gaps in discharge medication, follow-up, and cardiac rehabilitation adherence.^19^

## Healthcare policy and political factors

### National reimbursement systems

National reimbursement systems play a pivotal role in determining access to interventional cardiology procedures, particularly in the realm of costly structural interventions such as TAVI or transcatheter edge-to-edge repair (TEER). Restrictive reimbursement models, especially those that inadequately cover procedural costs or impose difficult approval processes can discourage hospitals from offering these therapies, even when they are clinically indicated. This issue is particularly pronounced in countries with budgetary constraints or fragmented payer systems, where multiple insurance or payment entities coexist. Furthermore, significant disparities arise between nations operating under universal healthcare frameworks, and those with privatized or mixed models. While universal systems may provide more equitable baseline access and preventive services across socioeconomic strata, they often face delays or restrictions in approving novel technologies, due to centralized budget controls and rationing. Conversely, privatized systems may offer quicker adoption in high-resource settings, but exacerbate inequities for underinsured or marginalized populations.^20^

### Regulatory hurdles

Regulatory frameworks are essential for ensuring patient safety and procedural efficacy, but excessive bureaucracy can slow the progress in interventional cardiology. Lengthy and complex approval processes for new devices or procedural authorizations often delay the introduction of innovative therapies, particularly in regions where regulatory agencies operate with limited resources. These delays can create significant disparities in patient access to cutting-edge interventions, with some countries experiencing years-long lags. While patient safety must remain paramount, a balanced approach is critical to ensuring that regulatory systems do not become barriers to equitable care delivery.^21,22^

### Political prioritization of cardiovascular care

The extent to which cardiovascular care is prioritized within a nation's political agenda has a profound influence on healthcare funding, infrastructure development, and policy implementation. Cardiovascular care often competes with oncology, infectious disease and mental health in budget prioritization. In countries where cardiovascular disease is recognized as a leading public health threat, strategic investments in prevention programs, specialized training, and interventional facilities are more common, leading to improved outcomes and system resilience. Conversely, in nations where political attention is directed elsewhere, often toward infectious diseases or more politically visible health crises, cardiovascular care may be underfunded and deprioritized.^23^

## Real-world clinical perspectives

### General challenges faced by physicians

Delivering interventional cardiovascular care is a highly complex endeavor shaped not only by clinical expertise but also by the structure and functionality of the healthcare system. Across different regions and systems, significant heterogeneity exists in how interventional cardiologists practice, with structural disparities influencing both patient outcomes and clinician well-being.

Interventional cardiologists frequently operate within health systems burdened by growing complexity, where barriers to optimal care extend beyond individual clinical judgment. These challenges are often rooted in broader systemic issues—such as bureaucratic inefficiencies, infrastructural limitations, and inequities in access to care. In both elective and emergent settings, physicians face obstacles such as delayed insurance preauthorizations, fragmented referral pathways, and a lack of integrated multidisciplinary teams or catheterization laboratory access—particularly outside major urban centers.

Time-sensitive cardiovascular conditions, such as STEMI and symptomatic aortic stenosis, require prompt diagnosis and intervention. However, reports across diverse healthcare systems indicate frequent delays due to administrative bottlenecks, geographic isolation, or inefficient patient transfer protocols.^24^ These delays are not benign: Observational data show that increased time from symptom onset to intervention is associated with significantly worse outcomes, including higher rates of heart failure, reinfarction, and mortality.^25^

The misalignment between ideal care and practical realities is especially pronounced in settings with constrained resources. Physicians are often forced to choose between the most evidence-based therapy and the most logistically feasible or financially viable alternative. This creates ethical dilemmas, particularly when high-value therapies are available but unattainable due to system constraints.^26^ The resulting cognitive dissonance contributes to what is increasingly recognized as *moral distress*—a condition where clinicians know the appropriate course of action but are unable to pursue it due to external limitations.^27^

### Physician testimonials

Growing literature underscores the psychological toll that systemic constraints exert on interventional cardiologists. Moral distress has been consistently associated with emotional exhaustion and burnout, particularly among clinicians in high-stakes, high-intensity environments such as acute cardiovascular care.^27,28^ A systematic review of 14 studies involving over 2000 healthcare professionals demonstrated a moderate-to-strong correlation between moral distress and measures of burnout (*r* ≈ 0.33).^29^

Common triggers include inability to access timely imaging or intervention, forced triage based on non-medical criteria such as insurance status, and lack of institutional support for guideline-based care. These factors contribute not only to professional dissatisfaction but may also impair clinical decision-making and increase turnover in an already stretched subspecialty workforce.^28,29^

First-hand accounts from interventional cardiologists frequently echo these findings. Clinicians report frustration at delayed STEMI transfers, limited access to transcatheter valve therapies, and ethical discomfort when choosing less effective treatments due to cost or availability. Burnout resulting from such chronic systemic pressures has implications for both physician well-being and patient safety.^29^

In recognition of these challenges, recent proposals have called for the integration of a dedicated ‘cardioethics’ curriculum within cardiology training. Such a framework aims to equip cardiologists with the ethical competencies needed to navigate the increasingly complex dilemmas they face. Key areas of focus include distributive justice, informed consent, and patient autonomy—principles that are essential in high-stakes, resource-variable environments. These ethical dimensions extend beyond individual patient encounters and intersect with broader issues of health equity, system design, and fairness in the allocation of care resources.^30^

### Comparative system analysis

Healthcare system structure plays a pivotal role in shaping access to interventional cardiology services. In the United States, advanced technologies and procedural expertise are widely available, but access is often hindered by fragmented insurance coverage, preauthorization delays, and referral barriers. High procedural volumes are concentrated in tertiary centers, but systemic inequities may delay or limit care, particularly for underinsured or high-risk patients.^31^

Conversely, the United Kingdom’s National Health Service (NHS) provides universal access, with more standardized referral and triage pathways. Emergency procedures such as primary PCI are generally well-coordinated and streamlined.^32^ However, capacity constraints, including cath lab backlogs and workforce shortages, may delay elective or complex structural interventions.^32^

In low- and middle-income countries (LMICs), limited infrastructure, workforce shortages, and supply chain interruptions are major impediments. Access is often restricted to urban centers, with rural populations underserved. Nonetheless, Non-Governmental Organizations (NGO)-supported programs, mobile cath labs, training exchanges, and telemedicine initiatives have improved procedural access and equity in certain regions.^33,34^

These system-level comparisons underscore the importance of health policy in enabling or constraining optimal cardiovascular care. Models that prioritize early access, minimize administrative delays, and invest in regional procedural capacity appear to yield the best outcomes.

## European union and WHO regulatory influence

### European union initiatives

Bridging disparities in interventional cardiology across the European Union (EU) remains a critical public health priority. Despite cardiovascular diseases (CVD) being the leading cause of death in Europe, access to advanced cardiovascular interventions varies significantly between Member States, particularly between Western and Eastern Europe.^35,36^ In Eastern European countries such as Romania and Bulgaria, the rates of PCI per million inhabitants are substantially lower compared with Western European countries like Germany or France.^37^ While healthcare systems remain largely under national control, the European Union has conducted different strategies (*Table 2*) to afford equitable access to cardiovascular interventions and to reduce health disparities across Member States.

**Table 2:** European initiatives to reduce health disparities in interventional cardiology across European members states^38,39^

EU Initiative	Definition	Year of Launch	Objectives	Key Actions	Examples/Impact	Impact on Bridging Disparities in Interventional Cardiology
**EAPCI Education and Training Grants** ^38^	Grants supporting 12-month clinical training in interventional cardiology for young cardiologists within ESC member countries.	Since ∼2007 (ongoing annually)	ESC National Cardiac Societies countries (majority of EU Member States and affiliated countries)	Develop skills and expertise of young interventional cardiologists to improve care quality.	Provide €25 000 grants for clinical fellowships at accredited European centers of excellence	Over 100 fellows trained since inception; enhances clinical skills, research experience, and professional networks.
**Stent For Life** ^39^	a European-led initiative (launched by the ESC/EAPCI, EuroPCR, and industry)	in 2009–2008	Aimed at improving rapid access to primary percutaneous coronary intervention—pPCI—particularly for STEMI patients worldwide.	Rapid adoption across Europe—Spain, Greece, Turkey, Czech Republic, etc.	Countries with previous low pPCI rates saw notable improvements; for example, Greece rose from 9 % to 32 % coverage within 2 years	Stent for Life, now Stent—Save a Life!, has catalyzed transformative change in STEMI care by systematically raising pPCI availability, reducing inequities, optimizing healthcare networks, and saving lives—through guideline adherence, coordinated health systems, and targeted training
**Valve For Life** ^40^	A European-led initiative (launched by the ESC/EAPCI, and industry)	Mid 2015 (ongoing)	aimed to improve transcatheter valve interventions (both aortic and mitral) across Europe—launched in four pilot countries: Poland, France, Portugal and United Kingdom (joined the initiave in 2020)	Raise awareness on the importance of valvular heart disease in the general population Facilitate access to novel therapies such as transcatheter heart valve interventions Raise educational standards, reduce obstacles to therapy implementation Diminish age and gender discrimination in access to care	Portugal: 2019 final results show a 73.9% increase in total valvular procedures per million inhabitants, and an 80% increase in TAVI procedures per million inhabitants, when compared with January 2017 numbers	The Valve for Life initiative has significantly increased the number of procedures—mainly TAVI, but over the years also TEER in the countries that participate in it, but it also raises awareness of valve diseases in general population
					Poland: increase in TAVI procedures (from 452 in 2014 to more than 1260 treatments in 2018); increase in MitraClip procedures form 6 procesures in 2010 to 186 procedures in 2019	
					France: *TAVI refunded by the authorities in France increased from 7 500 to 20 000 (+167%)*	
**EuroHeart Project** ^41^	Initiative to harmonize cardiovascular data collection and quality indicators across Europe for better evaluation and research.	∼2020	Standardize data and improve quality of cardiovascular care and clinical trials.	Develop unified registries, implement standardized metrics across countries.	Enables large-scale cardiovascular trials; improves comparability of care quality across EU Member States.	Facilitates equitable quality assessment and benchmarking of interventional cardiology outcomes across countries, identifying gaps and guiding improvements.
**EU4Health Program** (2021–2027)^42^	EU funding program aimed at strengthening health systems, improving disease prevention, and reducing health inequalities across Member States.	2021	Enhance healthcare infrastructure, reduce disparities in cardiovascular disease (CVD) care.	Fund projects Support digital health tools Promote screening and prevention programs.	Supports EuroHeart project; aims to reduce over 1.7 million annual CVD deaths and €282 billion economic burden in the EU.	Exp: the **Veneto Region Cardiovascular Screening Program (CARDIO50)** in Italy, which uses integrated risk assessments and digital tools to identify high-risk individuals over 50 years old, aiming to prevent cardiovascular events that may require interventional treatment. This model is being transferred to countries like Lithuania, Luxembourg, and Romania through the YOUNG50 project,
**Council Conclusions on Cardiovascular Health** ^43^	Policy framework promoting early detection, prevention, and equitable access to cardiovascular therapies across the EU.	2024	Promote screening, address social and environmental determinants, and improve care equity.	Encourage Member States to implement comprehensive cardiovascular health plans.	Commitment to an EU-wide cardiovascular health plan aiming to reduce premature deaths and health inequalities.	Sets political commitment and policy direction to prioritize reducing disparities, ensuring all Member States adopt measures to improve access and outcomes.

### World health organization (WHO) framework

The WHO has been working to mitigate disparities in interventional cardiology, as in other medical fields, notably through the creation of documents that provide guidance to political and organizational decision-makers, as well as to civil society. Examples include *The Framework for the Care of Acute Coronary Syndrome and Stroke*, the *WHO List of Priority Medical Devices for Management of Cardiovascular Diseases and Diabetes*, and the *World Report on Social Determinants of Health Equity* (Framework for the care of acute coronary syndrome and stroke. Geneva: World Health Organization; 2024. Licence: CC BY-NC-SA 3.0 IGO. ISBN 978–92-4-010366-5), [WHO list of priority medical devices for management of CVD and diabetes. Geneva: World Health Organization; 2021(WHO medical device technical series). Licence: CC BY-NC-SA 3.0 IGO. ISBN 978-92-4-002797-8], (World report on social determinants of health equity. Geneva: World Health Organization; 2025. Licence: CC BY-NC-SA 3.0 IGO. ISBN 978-92-4-010758-8). These aim to ensure that, despite the socioeconomic gap between different countries, there is a similar goal of delivering equitable healthcare services.

Since 2023, the WHO has made available the *Health Inequality Data Repository*, accessible through the *HEAT (Health Equity Assessment Tool)*—an interactive platform that hosts the largest health-related (and beyond) database. Its purpose is to make inequality visible and measurable, providing information on disparities in behaviors, pathologies, and outcomes across different countries, age groups, gender, education levels, economic status, and other dimensions of inequality. The data are available through the WHO Health Inequality Monitor^44^ and the HEAT (Health Equity Assessment Toolkit),^45^ which offer access to structured datasets and tools supporting the development of equity-focused health programs.^46^ Additionally, the WHO has been developing methodologies for quantifying health inequality, such as by, for example, the document *Health Inequity Monitoring*.^44^

Similarly, the *World Heart Federation* also makes clear—through its *World Heart Vision 2030: Driving Policy Change*—its commitment to identifying inequalities and addressing change strategies aimed at improving cardiovascular health.^47^

### Patient awareness, education, and public campaigns

A critical yet frequently underestimated determinant of disparities in interventional cardiology is patient awareness and health literacy.^48^ Socioeconomic, geographic, and political inequalities are often magnified by an individual’s limited ability to recognize cardiovascular risk or symptoms, even when structural improvements in access to care are achieved. Health care providers regularly encounter patients who present too late for optimal interventions, not due to system failures alone, but because they were unaware that occurring symptoms like chest pain, dyspnea, or fatigue could signify a life-threatening.

### Health literacy and cardiovascular risk recognition

Patients from socioeconomically disadvantaged backgrounds consistently show lower levels of health literacy. Numerous studies have confirmed that these individuals are significantly less likely to identify symptoms of acute coronary syndromes or appreciate the urgency of seeking care, often resulting in missed windows for reperfusion therapies such as primary PCI.^49^ Meta-analyses further demonstrate that low health literacy is independently associated with increased cardiovascular mortality, poorer adherence to therapy, and higher readmission rates.^48,50^ In women from disadvantaged communities, low cardiovascular health literacy is also linked to poor patient–physician communication.^51^ Additionally, digital literacy disparities, especially among older adults and marginalized groups, can further limit access to educational resources and telehealth interventions.^52^

### Role of social and public health campaigns

Public education campaigns are a powerful, still underutilized, tool to improve cardiovascular outcomes. Programs like ‘*Don’t Die of Doubt’* and ‘*Minutes Matters*’ by the American Heart Association but also EU-funded rural outreach, and localized community initiatives have shown measurable improvements in early symptom recognition and hospital presentation.^53^ Campaigns using behaviorally informed strategies, such as priming, have demonstrated added efficacy in reinforcing cardiovascular risk awareness.^54^ However, many of these efforts lack cultural adaptation, continuity, or sufficient funding. To maximize impact, campaigns must be sustained, multilingual, and co-developed with communities to reflect local realities and beliefs.^55,56^ Educating and empowering patients is essential not only to reduce procedural delays but to enhance long-term cardiovascular outcomes across all population strata. Collaboration with patient advocacy organizations worldwide has the potential to drive the most meaningful and lasting impact. Within the European Society of Cardiology (ESC), this effort is supported by the ESC Patient Forum, which brings together patient representatives from across Europe^57^

## Bridging the gap: solutions and future directions

Addressing disparities in access to interventional cardiology requires a multifaceted approach that combines technological innovation, workforce development, and policy reform.

### Telemedicine and remote diagnostics

Telemedicine has emerged as a transformative tool to overcome geographic barriers and enhance access to specialized cardiac care. Progress has already been made by using telemedicine for monitoring patients and guiding minor therapeutic decisions, such as up-titrating medication dosages, in conditions like heart failure, atrial fibrillation, and hypertension. There is also a growing global trend toward the development of advanced telemonitoring biosensors and implantable devices delivered via transcatheter procedures.^58^ Emerging technologies such as AI-enabled diagnostic platforms, consumer wearables, portable ECG monitors, and smartphone-based applications are increasingly recognized as potential equalizers in cardiovascular care. With large smartphone penetration,^18^ these tools offer unprecedented opportunities for early detection of arrhythmias, continuous rhythm or hemodynamic monitoring, and timely referral in both high- and low-resource settings. Studies have already demonstrated the feasibility of photoplethysmography- and ECG-based wearables for atrial fibrillation screening and STEMI detection, with clinically meaningful diagnostic accuracy.^59,60^ Moreover, virtual consultations play a key role in facilitating early triage for patients with urgent cardiac conditions, particularly in rural and underserved areas. By enabling remote assessment, telehealth platforms ensure timely referrals to interventional centers when needed, reducing delays and optimizing care pathways. Additionally, the use of mobile units equipped with point-of-care diagnostic tools, can help identify patients with critical needs, such as those with severe aortic stenosis or acute coronary syndromes, facilitating access to advanced interventional care into remote communities. Clinical studies have consistently demonstrated the reliability and effectiveness of telemedicine technologies in improving patient outcomes and quality of life. Moreover, these approaches have shown the potential to enhance cost-effectiveness from the perspective of healthcare systems.^61^ Nevertheless, critical challenges remain data security, confidentiality and reimbursement models, which must be addressed to ensure sustainable and equitable integration into routine practice.^62^

Recent successful examples of telemedicine application in interventional cardiology include STEMI networks like the Mission: Lifeline STEMI Systems Accelerator in the U.S., leveraging on remote ECG transmission and teleconsultation to enable early cath lab activation, reducing door-to-balloon times and improving outcomes.^24^ Post-PCI follow-up via telemonitoring has shown promise in trials like TIM-HF2, where structured remote management led to lower mortality in high-risk patients, many of whom had recent coronary interventions.^61^ In structural heart disease, virtual heart teams were successfully implemented during the COVID-19 pandemic in Italy and France, allowing remote case discussion and imaging review for TAVI and MitraClip candidates without delaying care.^63^ In resource-limited regions such as India and Brazil, ‘teleproctoring’ allowed senior interventionalists to remotely guide complex procedures—including CTO PCI and valve-in-valve cases—via live video and fluoroscopy feeds.^64^ Additionally, mobile cath labs deployed in rural China and parts of Africa have been supported by teleconsultation from tertiary centers, enabling diagnostic and elective interventional procedures in areas lacking fixed facilities.^34^ These experiences underscore the potential of telemedicine to decentralize interventional cardiology, support clinician training, and extend timely, high-quality care to remote populations.

### Decentralized training and resource allocation

Building an effective interventional cardiology network requires targeted investments in decentralized training and workforce development. The establishment of regional centers of excellence for clinical training, mentorship, and procedural support, has the potential to significantly strengthen capacity in underserved areas. These centers can act as hubs, ensuring that local providers have access to the latest techniques and best practices in the field of interventional cardiology. Simultaneously, incentive programs such as scholarships, loan forgiveness, or competitive salaries can motivate healthcare professionals to pursue training in interventional cardiology and to commit to practicing in rural or resource-limited environments. Building local expertise in percutaneous interventions fundamentals and hands-on procedural skills is essential to establishing a sustainable, equitable network of cardiovascular care that can reach all patients, regardless of their location.^63,64^ An essential component of reducing disparities in acute cardiac care is the active involvement of paramedics in both diagnostic and therapeutic pathways.^65^ Evidence from the U.S. and Europe shows that empowering paramedics to perform and transmit pre-hospital ECGs, initiate triage, and administer first-line therapy reduces delays in STEMI treatment and improves outcomes. Building on this, paramedic-focused education programs have been shown to increase the proportion of direct STEMI transfers to PCI-capable centers and significantly reduce door-to-balloon times, thereby enhancing system efficiency and patient outcomes.^66,67^

### Policy advocacy and reimbursement reform

International cardiology societies and professional associations play a pivotal role in fostering dialogue with legislators and health authorities to support equitable access to advanced cardiovascular interventions.^68^ This includes efforts to simplify regulatory processes, streamline approvals for devices and procedures, and reduce administrative burdens that delay timely treatment. Reimbursement models must also evolve. Current fee-for-service systems may inadvertently discourage hospitals from offering costly structural interventions, especially in low-resource settings. Promoting value-based care models that reward outcomes rather than procedural volume could help align financial incentives with patient-centered goals, improving access to advanced interventional therapies while ensuring cost-effectiveness.

## Conclusion

Disparities in interventional cardiology affect both patients and healthcare professionals. Unequal access to procedures like coronary and structural interventions leads to avoidable delays, worse outcomes, and deepens existing health inequities. At the same time, clinicians working in underserved or resource-limited settings face professional isolation, moral distress, and burnout—consequences of being unable to deliver guideline-based care due to systemic barriers. These inequities compromise not only care delivery but also workforce sustainability and morale. Bridging these gaps requires coordinated action to improve infrastructure, reimbursement models, workforce distribution, and policy frameworks—ensuring equitable, high-quality cardiovascular care for all and supporting those who deliver it. A future where interventional cardiology is accessible to all is not only desirable but achievable—if equity becomes a shared clinical and political imperative.

## Lead author biography


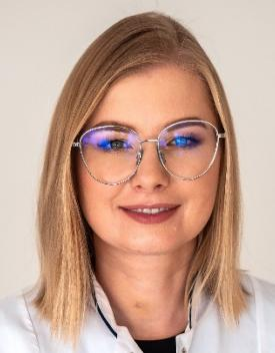


Associate Prof. Marta Kałużna-Oleksy is an interventional cardiologist at the University of Medical Sciences in Poznań, Poland. Her research focuses on acute and chronic coronary syndrome, cardiogenic shock, and sex differences in cardiovascular diseases. Associate Prof. Kałużna-Oleksy is Chair of Gender and Disparities Committee of European Association of Percutaneous Interventions (2024–2026) and Board Member of Polish Association of Percutaneous Cardiovascular Interventions (2023–2025, 2025–2027) and President-elect of Polish Association of Acute Cardiac Care (2025–2027) and actively promotes patient-centered care, interdisciplinary collaboration, and female leadership in cardiology.

## Data Availability

No new data were generated or analysed in support of this research.
